# Supplementary material for: β-Catenin in Dendritic Cells Negatively Regulates CD8 T Cell Immune Responses through the Immune Checkpoint Molecule Tim-3
Source: Vaccines (Basel). 2024 Apr 25;12(5):460. doi: 10.3390/vaccines12050460 (PMC11125945; doi:10.3390/vaccines12050460)
Supplement: Supplementary file 1 [file vaccines-12-00460-s001.zip › vaccines-2943877-supplementary.pdf]

## Supplemental Figures

## Supplemental Figure S1

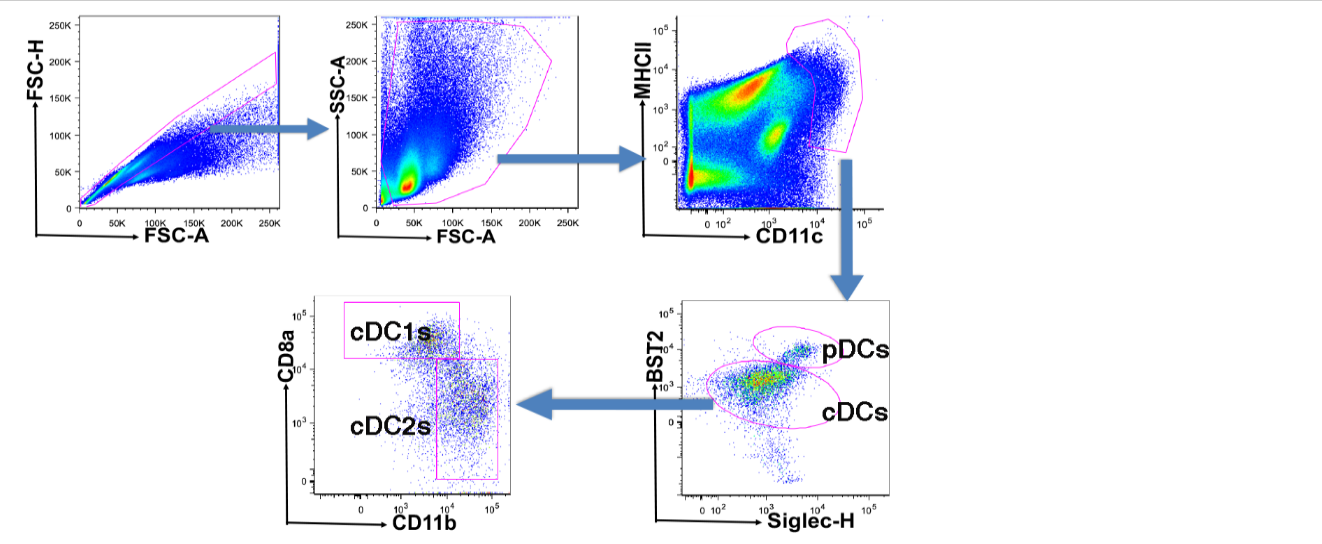


## Supplemental Figure S2

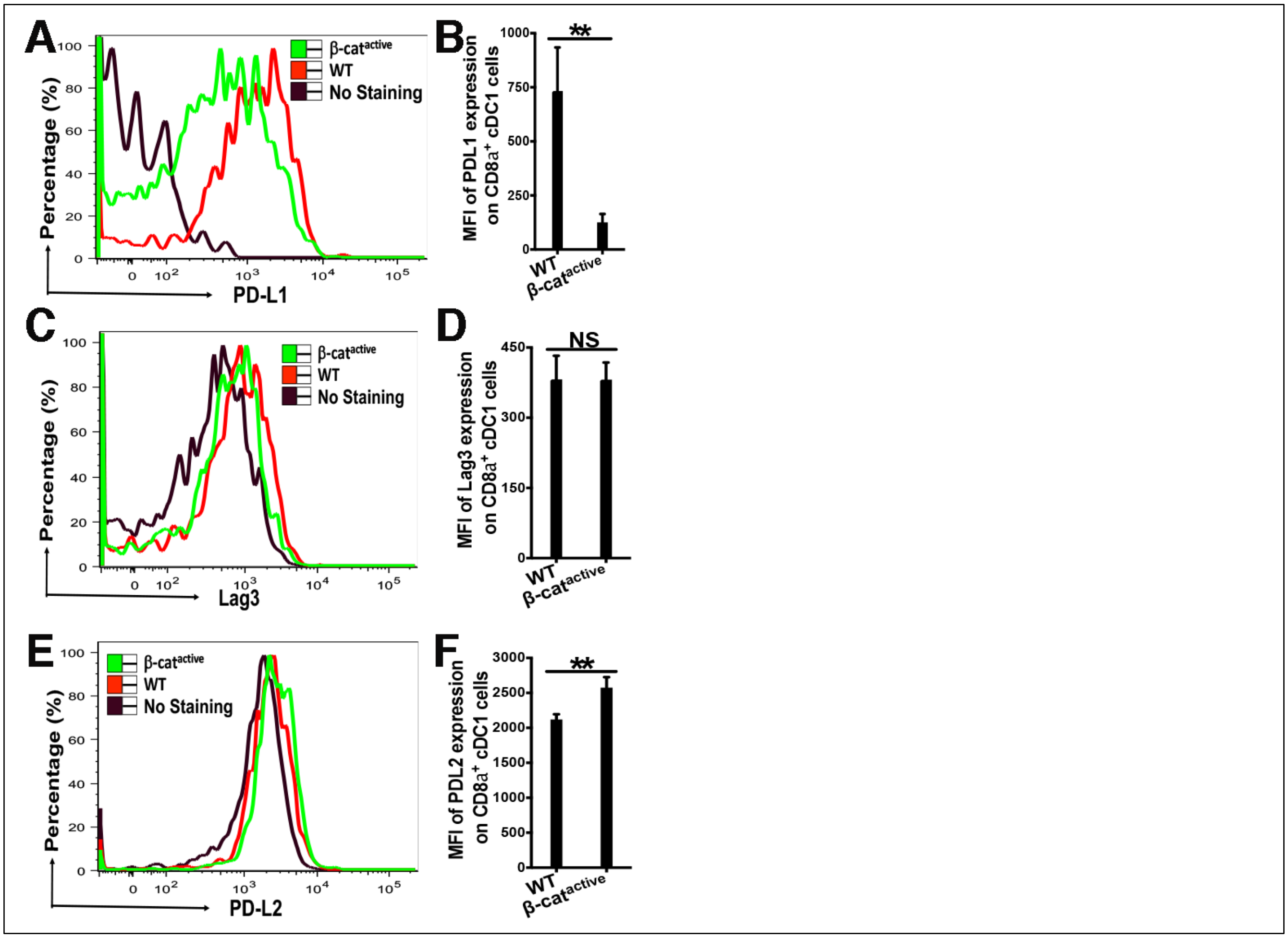

## Supplemental Figure S3

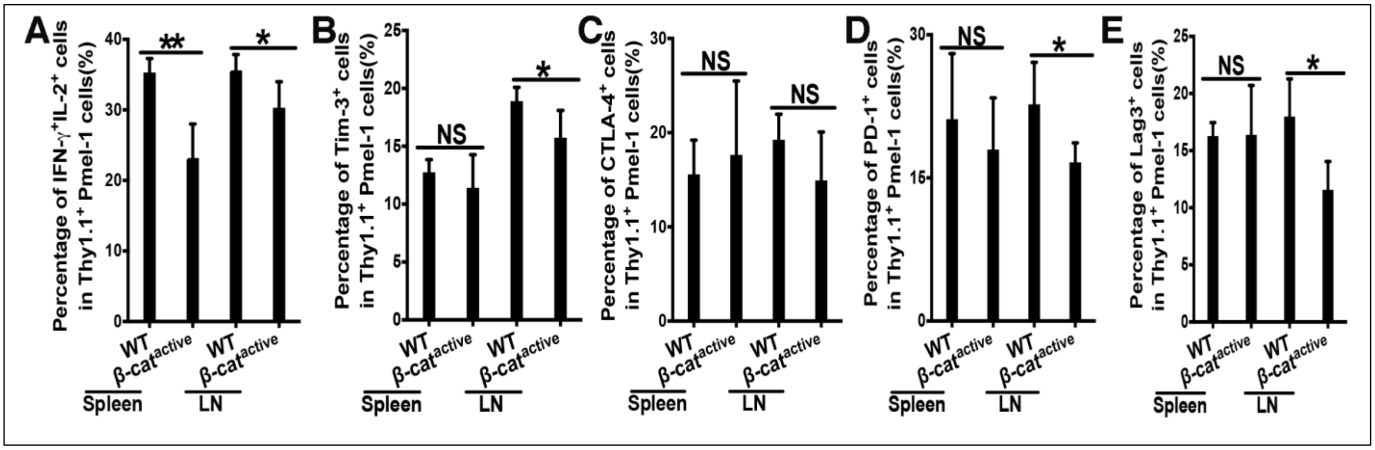


## Supplemental Figure S4

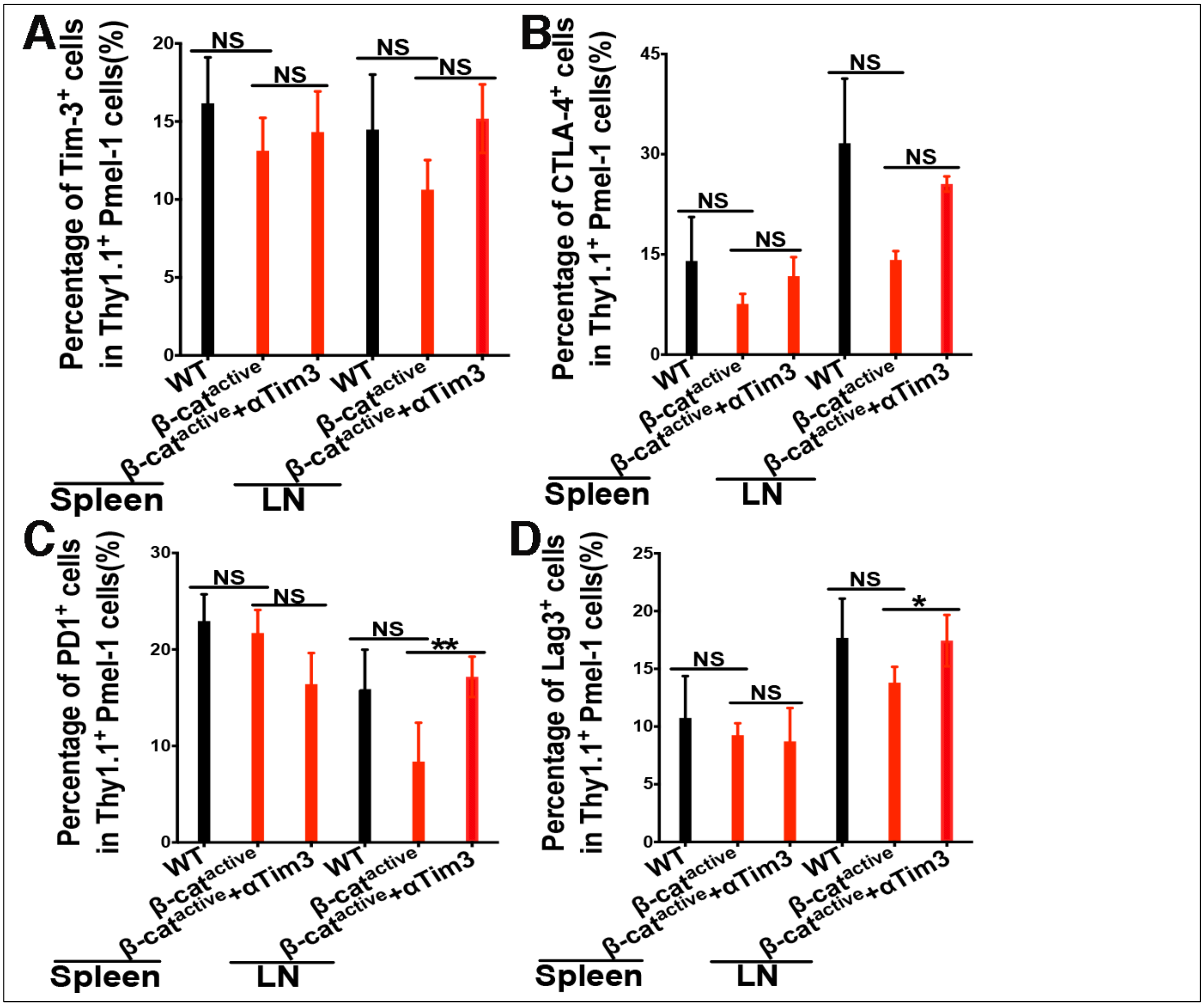

## Supplemental Figure Legends

**Figure S1. Gating strategy for splenic DCs.**

**Figure S2. The effects of β-catenin on expression of inhibitory molecules PD-L1, PD-L2 and Lag3 in splenic cDC1s.** Splenic cells from WT and CD11c-β-catenin$^{active}$ mice (n=4) were stained and analyzed by flow cytometry, and the expression of inhibitory molecules PD-L1 (A-B), Lag3 (C-D), and PD-L2 (E-F) on gated CD8α$^{+}$ cDC1s were shown as in Figure 1. Student's t test, $^{***}P < 0.001$, $^{**}P < 0.01$, $^{*}P < 0.05$, and NS > 0.05. Data shown are representative of three experiments.

**Figure S3. β-catenin in DCs negatively regulates DC vaccine-cross-priming against tumor antigen.** WT and CD11c-β-catenin$^{active}$ mice (n=5) were immunized with anti-DEC-205-hgp100 plus CpG, and cross-priming of adoptively transferred Thy1.1$^{+}$ Pmel-1 CD8 T cells was examined at day 5 following 5 hour *in vitro* stimulation with hgp100$_{25-33}$ with Brefeldin A (BFA). The percentages of IFN-γ$^{+}$IL-2$^{+}$ **(A)**, Tim-3$^{+}$ **(B)**, CTLA-4$^{+}$ **(C)**, PD-1$^{+}$ **(D)**, and Lag3$^{+}$ **(E)** cells out of total Thy1.1$^{+}$ Pmel-1 cells are shown. Student's t test, $^{***}P < 0.001$, $^{**}P < 0.01$, $^{*}P < 0.05$, and NS > 0.05. Data are representative of three experiments.

**Figure S4. Anti-Tim-3 treatment reverses β-catenin-mediated inhibition of cross-priming.** WT and CD11c-β-catenin$^{-/-}$ mice (n=4-5) were immunized with anti-DEC-205-hgp100 plus CpG with or without anti-Tim-3 treatment, and cross-priming of adoptively transferred Thy1.1$^{+}$ Pmel-1 CD8 T cells was examined at day 5 after immunization. The percentages of Tim-3$^{+}$ (A),

CTLA-4$^+$ (B), PD-1$^+$ (C), and Lag3$^+$ (D) Pmel-1 cells in total Thy1.1$^+$ Pmel-1 cells are shown. Data are representative of two experiments. . One-way ANOVA and Post-hoc T tests with Bonferroni correction were used. $^{***}P < 0.001$, $^{**}P < 0.01$, $^{*}P < 0.05$ and NS > 0.05.
